# Crystal structure of Rb_6_[B_12_O_18_(OH)_6_]·2H_2_O

**DOI:** 10.1107/S2056989022008611

**Published:** 2022-09-02

**Authors:** Qi-Ming Qiu, Li Yan, Jian-Biao Song

**Affiliations:** a School of Science, China University of Geosciences, Beijing 100083, People’s Republic of China; b Analytical and Testing Centre, Beijing Institute of Technology, Beijing 100081, People’s Republic of China; c Beijing Chaoyang Foreign Language School, Beijing 100101, People’s Republic of China; University of Neuchâtel, Switzerland

**Keywords:** alkaline metal borate, solvothermal synthesis, hydrogen bond, supra­molecular framework, crystal structure

## Abstract

The solvothermal reaction of H_3_BO_3_, sodium *tert*-butoxide, Rb_2_CO_3_ and pyridine led to a new alkaline metal borate hexa­rubidium hexa­hydroxy­dodeca­borate dihydrate, Rb_6_[B_12_O_18_(OH)_6_]·2H_2_O. Its structure contains a large cyclic dodeca­oxoboron cluster, [B_12_O_18_(OH)_6_]^6−^, formed by six {B_3_O_3_} rings. In the crystal, O—H⋯O hydrogen bonds between the components lead to the formation of a three-dimensional supra­molecular framework.

## Abstract 2

The solvothermal reaction of H_3_BO_3_, sodium *tert*-butoxide, Rb_2_CO_3_ and pyridine led to a new alkaline metal borate hexa­rubidium hexa­hydroxy­dodeca­borate dihydrate. Its structure contains a large cyclic dodeca­oxoboron cluster, [B_12_O_18_(OH)_6_]^6−^, formed by six {B_3_O_3_} rings. In the crystal, O—H⋯O hydrogen bonds between the components lead to the formation of a three-dimensional supra­molecular framework.

## 1. Chemical context

In recent years, borates have made excellent contributions to the development of nonlinear optical (NLO) materials and so they are the focus of material chemists (Bashir *et al.*, 2018 ▸; Qiu *et al.*, 2021*a* ▸; Wei *et al.*, 2016 ▸). Scientists have found that alkali- and alkaline-earth–metal borates often exhibit a short ultraviolet cut-off edge due to no *d*–*d* and *f*–*f* electron transition in the ultraviolet region with wide transparency ranges (Shi *et al.*, 2019 ▸; Tang *et al.*, 2019 ▸). Generally, boron has two kinds of coordination modes: either BO_3_ trigonal or BO_4_ tetra­hedral, and they further bond to each other through common O atoms forming different oxoboron clusters, which can further polymerize into isolated clusters, one-dimensional chains, two-dimensional layers or three-dimensional frameworks. Here, single crystals of Rb_6_[B_12_O_18_(OH)_6_]·2H_2_O with alkali metal atoms and isolated oxoboron clusters have been obtained under solvothermal conditions. 
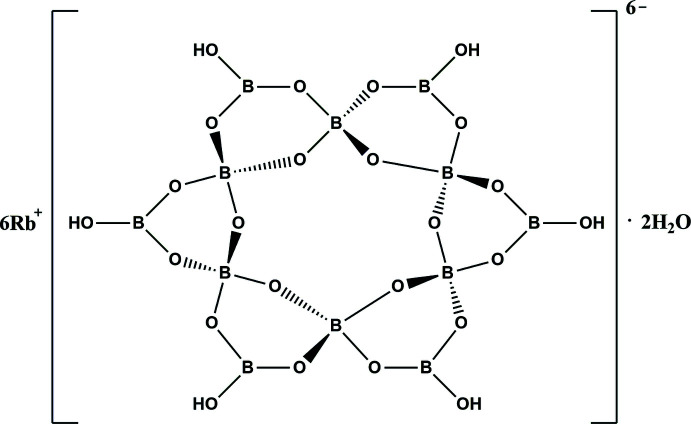


## 2. Structural commentary

**Figure 1:**
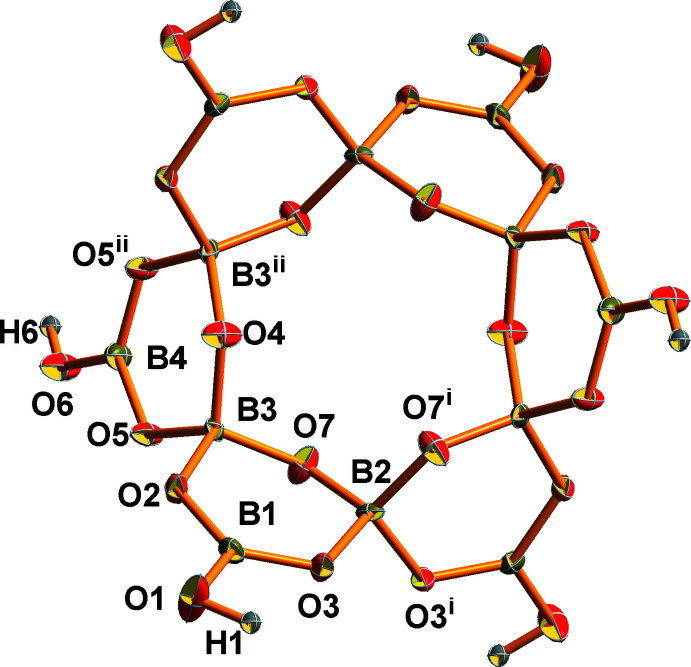
The asymmetric unit of the oxoboron cluster of [B_12_O_18_(OH)_6_]^6−^ [symmetry codes: (i) 2 − *x*, 2 − *y*, *z*; (ii) *x*, *y*, 2 − *z*]. Displacement ellipsoids are drawn at the 50% probability level.

There are 13.5 independent atoms in the asymmetric unit of the title compound, including 3 B, 9/2 O, 3/2 OH, 3/2 Rb, and 1/2 H_2_O. It should be noted that the Rb1, Rb2, B2, B4, O4, O6 and O8 atoms are located on special positions with occupancy of 0.25 or 0.5, while the remaining Rb, B and O atoms are located at general positions with an occupancy of 1. Bond-valence-sum calculations show that Rb and B are consistent with the expected oxidation states (Brown & Altermatt, 1985 ▸; Brese & O’Keeffe, 1991 ▸). Six trigonal BO_2_(OH) units [B—O(av.) = 1.360 Å] and six tetra­hedral BO_4_ units [B—O(av.) = 1.474 Å] are linked by vertex sharing. Each BO_4_ unit provides two terminal oxygen atoms to connect with two neighboring BO_4_ units and shares the other two corners with the BO_2_(OH) unit to form a [B_12_O_18_(OH)_6_]^6−^ cluster (Fig. 1 ▸). Each Rb atom is six-coordinate, with Rb—O distances in the range of 2.793 (5)-3.359 (5) Å.

## 3. Supra­molecular features

**Figure 2:**
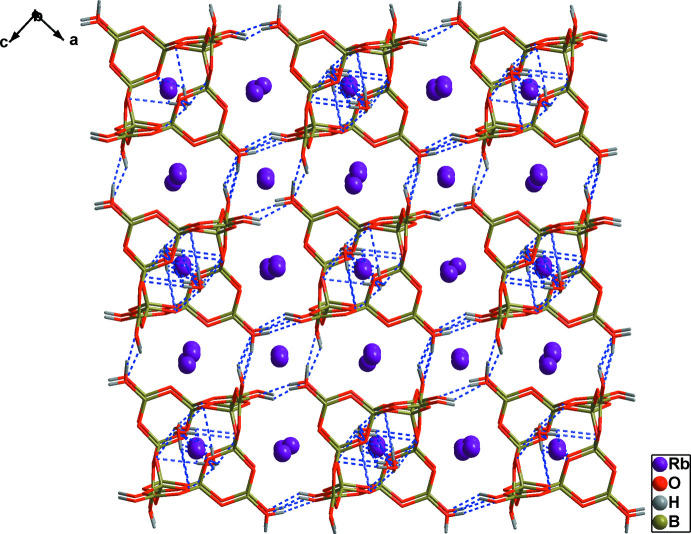
View of the three-dimensional supra­molecular framework along the [010] direction. All of the Rb—O bonds are omitted for clarity and blue dashed lines represent O—H⋯O hydrogen bonds.

In the title compound, each [B_12_O_18_(OH)_6_]^6−^ cluster is connected to other clusters by O1—H1⋯O6, and O6—H6⋯O1 hydrogen bonds, resulting in a three-dimensional supra­molecular framework (Fig. 2 ▸, Table 1 ▸). Water mol­ecules are also attached to supra­molecular structure *via* O—H⋯O hydrogen bonds. The title structure is different from those of previously reported analogues K_7_{(BO_3_)Mn[B_12_O_18_(OH)_6_]}·H_2_O (Zhang *et al.*, 2004 ▸), and Na_2_Cs_4_Ba_2_[B_12_O_18_(OH)_6_]·4OH (Zhang *et al.*, 2016 ▸). Both compounds crystallize in the non-centrosymmetric *Pmn*2_1_ space group and their supra­molecular structures are different from that of the title compound. Therefore, the use of different alkali metals as templates may affect the crystallization of the oxoboron supra­molecular structure.

## 4. Database survey

A search of the Cambridge Structural Database (CSD, version 5.43, update June 2022; Groom *et al.*, 2016 ▸) for the cyclic dodeca-oxoboron unit {B_12_O_24_} ring gave eight hits. In the crystals of Li_7_Na_2_KRb_2_B_12_O_24_, Li_7.35_Na_2.36_K_1.50_Cs_0.78_B_12_O_24_, Li_6.97_Na_2.63_K_1.24_Cs_1.15_B_12_O_24_, and Li_7.27_Na_2.67_Rb_2.06_B_12_O_24_ (refcodes: JOGBIT, JOGBOZ, JOFNEA, JOFNIE, trigonal, *R* 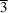 space group; Baiheti *et al.*, 2019 ▸), the terminal oxygens of this type of the {B_12_O_24_} ring can be completely deprotonated [B_12_O_24_]^12−^ and fail to extend to high-dimensional structures through covalent bonds and hydrogen bonds. In the crystal of Na_8_[B_12_O_20_(OH)_4_] (refcode: ETIJAU, monoclinic, *P*2_1_/*c* space group; Menchetti *et al.*, 1979 ▸), the partially protonated [B_12_O_20_(OH)_4_]^8−^ unit also fails to extend to a higher dimensional structure through O—B—O bonds. While KNa_8_[Li@B_12_O_18_(OH)_6_](CO_3_)_2_ (refcode: EBUCAJ, trigonal, *R* 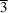 space group; Qiu *et al.*, 2021*b* ▸) is a borate carbonate with the isolated [Li@B_12_O_18_(OH)_6_]^5−^ cluster and inter­esting layers formed by Na^+^ and CO_3_^2−^ ions, thus forming a two-dimensional supra­molecular structure. After changing the synthetic conditions, the isolated [Li@B_12_O_18_(OH)_6_]^5−^ cluster was successfully extended to a layered structure *via* B—O—B bonds in Cs_5_[Li@B_12_O_20_(OH)_2_]·3H_2_O (refcode: EBUCIR, monoclinic, *Pc* space group; Qiu *et al.*, 2021*b* ▸), by condensation reactions with the elimination of water mol­ecules between oxoboron clusters.

## 5. Synthesis and crystallization

**Figure 3:**
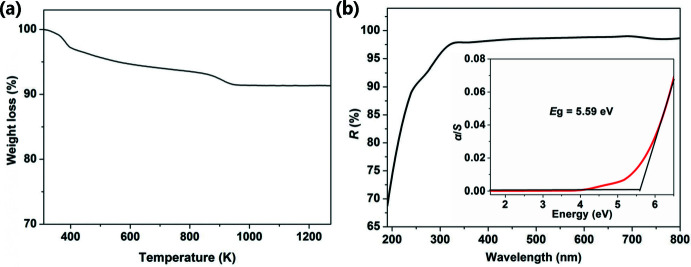
(*a*) Thermogravimetric curve and (*b*) ultraviolet visible diffuse reflectance spectrum of the title compound. Inset: plots of α/*S versus E*.

A mixture of H_3_BO_3_ (0.618 g, 10 mmol), sodium *tert*-butoxide (0.096 g, 1 mmol) and Rb_2_CO_3_ (0.231 g, 1 mmol) was added into pyridine (3.0 mL). After stirring for 15 min, the resulting mixture was sealed in a 25 mL Teflon-lined stainless steel autoclave, heated at 483 K for 7 days, and then slowly cooled to room temperature. Colorless block-shaped crystals of Rb_6_[B_12_O_18_(OH)_6_]·2H_2_O were obtained (yield 51% based on H_3_BO_3_). Infrared (KBr pallet, cm^−1^): 3445*vs*, 1639*m*, 1427*s*, 1320*m*, 1003*m*, 939*w*, 873*m*, 721*m*, 622*w*, 542*m*. The thermogravimetric curve of the title compound is shown in Fig. 3 ▸ *a*. The weight loss of 8.6% (cal. 8.4%) in the temperature range 350–950 K for the compound is attributed to the loss of the water mol­ecules and the removal of dehydration of the hydroxyl groups. The compound has almost no weight loss after 950 K. The ultraviolet visible diffuse reflectance spectrum of the title compound is shown in Fig. 3 ▸ *b*. The band gap obtained by extrapolating the linear part of the rising curve to zero for the compound is 5.59 eV.

## 6. Refinement

Crystal data, data collection and structure refinement details are summarized in Table 2 ▸. Hydrogen-atom coordinates were refined without any constraints or restraints. Their *U*_iso_ values were set to 1.2*U*_eq_ of the parent atoms.

## Supplementary Material

Crystal structure: contains datablock(s) I, global. DOI: 10.1107/S2056989022008611/tx2056sup1.cif

Structure factors: contains datablock(s) I. DOI: 10.1107/S2056989022008611/tx2056Isup3.hkl

CCDC reference: 2192069

Additional supporting information:  crystallographic information; 3D view; checkCIF report

## Figures and Tables

**Table 1:** Hydrogen-bond geometry (Å, °)

*D*—H⋯*A*	*D*—H	H⋯*A*	*D*⋯*A*	*D*—H⋯*A*
O8—H8*B*⋯O7^i^	0.85	2.25	3.046 (6)	155
O8—H8*B*⋯O4^i^	0.85	1.68	2.224 (7)	119
O8—H8*A*⋯O7^ii^	0.85	1.70	2.231 (5)	118
O8—H8*A*⋯O4^ii^	0.85	2.17	2.958 (7)	155
O6—H6⋯O1^iii^	0.82	1.86	2.670 (5)	167
O1—H1⋯O6^iv^	0.94	1.91	2.670 (5)	136

Symmetry codes: (i) 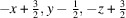 ; (ii) 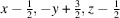 ; (iii) 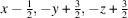 ; (iv) 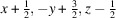 .

**Table 2:** Experimental details

Crystal data	
Chemical formula	Rb_6_[B_12_O_18_(OH)_6_]·2H_2_O
*M*_r_	1068.62
Crystal system, space group	Orthorhombic, *P* *n* *n* *m*
Temperature (K)	296
*a*, *b*, *c* (Å)	13.395 (4), 9.251 (2), 12.368 (4)
*V* (Å^3^)	1532.7 (7)
*Z*	2
Radiation type	Mo *K*α
μ (mm^−1^)	9.60
Crystal size (mm)	0.08 × 0.07 × 0.07

Data collection	
Diffractometer	Bruker APEXII CCD
Absorption correction	Multi-scan (*SADABS*; Krause *et al.*, 2015 ▸)
*T*_min_, *T*_max_	0.452, 0.746
No. of measured, independent and observed [*I* > 2σ(*I*)] reflections	17510, 1980, 1523
*R*_int_	0.057
(sin θ/λ)_max_ (Å^−1^)	0.667

Refinement	
*R*[*F* ^2^ > 2σ(*F* ^2^)], *wR*(*F* ^2^), *S*	0.061, 0.173, 1.07
No. of reflections	1980
No. of parameters	110
H-atom treatment	H-atom parameters constrained
Δρ_max_, Δρ_min_ (e Å^−3^)	1.57, −1.16

Computer programs: *APEX2* and *SAINT* (Bruker, 2014 ▸), *SHELXT2018/3* (Sheldrick, 2015*a* ▸), *SHELXL2018/3* (Sheldrick, 2015*b* ▸) and *SHELXTL* (Sheldrick, 2008 ▸).

## supplementary crystallographic information

### Crystal data

**Table:**

Rb_6_[B_12_O_18_(OH)_6_]·2H_2_O	*D*_x_ = 2.316 Mg m^−^^3^
*M**_r_* = 1068.62	Mo *K*α radiation, λ = 0.71073 Å
Orthorhombic, *P**n**n**m*	Cell parameters from 3469 reflections
*a* = 13.395 (4) Å	θ = 2.7–26.1°
*b* = 9.251 (2) Å	µ = 9.60 mm^−^^1^
*c* = 12.368 (4) Å	*T* = 296 K
*V* = 1532.7 (7) Å^3^	Block, colorless
*Z* = 2	0.08 × 0.07 × 0.07 mm
*F*(000) = 1000	

### Data collection

**Table:**

Bruker APEXII CCD diffractometer	1523 reflections with *I* > 2σ(*I*)
Radiation source: fine-focus sealed tube, Bruker (Mo) X-ray Source	*R*_int_ = 0.057
φ and ω scans	θ_max_ = 28.3°, θ_min_ = 2.2°
Absorption correction: multi-scan (SADABS; Krause *et al.*, 2015)	*h* = −17→17
*T*_min_ = 0.452, *T*_max_ = 0.746	*k* = −12→12
17510 measured reflections	*l* = −16→16
1980 independent reflections	

### Refinement

**Table:**

Refinement on *F*^2^	0 restraints
Least-squares matrix: full	Hydrogen site location: difference Fourier map
*R*[*F*^2^ > 2σ(*F*^2^)] = 0.061	H-atom parameters constrained
*wR*(*F*^2^) = 0.173	*w* = 1/[σ^2^(*F*_o_^2^) + (0.0825*P*)^2^ + 9.4675*P*] where *P* = (*F*_o_^2^ + 2*F*_c_^2^)/3
*S* = 1.07	(Δ/σ)_max_ < 0.001
1980 reflections	Δρ_max_ = 1.57 e Å^−^^3^
110 parameters	Δρ_min_ = −1.16 e Å^−^^3^

### Special details

**Table:**

Geometry. All esds (except the esd in the dihedral angle between two l.s. planes) are estimated using the full covariance matrix. The cell esds are taken into account individually in the estimation of esds in distances, angles and torsion angles; correlations between esds in cell parameters are only used when they are defined by crystal symmetry. An approximate (isotropic) treatment of cell esds is used for estimating esds involving l.s. planes.

### Fractional atomic coordinates and isotropic or equivalent isotropic displacement parameters (Å^2^)

**Table:**

	*x*	*y*	*z*	*U*_iso_*/*U*_eq_	Occ. (<1)
Rb1	1.000000	1.000000	0.500000	0.0418 (4)	
Rb2	1.000000	0.500000	1.000000	0.0529 (5)	
Rb3	0.74535 (5)	1.03106 (9)	0.73060 (7)	0.0489 (3)	
O1	1.0385 (4)	0.6325 (4)	0.6768 (4)	0.0397 (12)	
H1	1.089080	0.687977	0.643718	0.048*	
O2	0.9347 (3)	0.7048 (4)	0.8193 (3)	0.0191 (8)	
O3	1.0311 (3)	0.8776 (4)	0.7204 (3)	0.0203 (8)	
O4	0.9428 (4)	0.7903 (6)	1.000000	0.0204 (11)	
O5	0.7857 (3)	0.7944 (4)	0.9032 (3)	0.0194 (8)	
O6	0.6357 (4)	0.7801 (7)	1.000000	0.0270 (13)	
H6	0.599457	0.797673	0.948224	0.032*	0.5
O7	0.9137 (3)	0.9603 (4)	0.8563 (3)	0.0224 (8)	
O8	0.4074 (4)	0.3941 (5)	0.500000	0.0144 (9)	
H8A	0.401585	0.483846	0.486660	0.017*	0.5
H8B	0.456785	0.383526	0.542470	0.017*	0.5
B1	0.9999 (4)	0.7413 (6)	0.7399 (4)	0.0193 (11)	
B2	1.000000	1.000000	0.7919 (6)	0.0127 (14)	
B3	0.8956 (4)	0.8163 (6)	0.8960 (4)	0.0114 (10)	
B4	0.7386 (6)	0.7897 (9)	1.000000	0.0176 (15)	

### Atomic displacement parameters (Å^2^)

**Table:**

	*U*^11^	*U*^22^	*U*^33^	*U*^12^	*U*^13^	*U*^23^
Rb1	0.0586 (9)	0.0472 (8)	0.0196 (6)	0.0159 (6)	0.000	0.000
Rb2	0.0971 (13)	0.0268 (6)	0.0348 (7)	0.0271 (7)	0.000	0.000
Rb3	0.0324 (4)	0.0516 (5)	0.0628 (5)	0.0101 (3)	−0.0090 (3)	0.0248 (4)
O1	0.059 (3)	0.0192 (19)	0.041 (3)	−0.011 (2)	0.036 (2)	−0.0119 (18)
O2	0.0257 (18)	0.0140 (16)	0.0175 (18)	−0.0067 (14)	0.0084 (15)	−0.0047 (13)
O3	0.0296 (18)	0.0153 (16)	0.0158 (17)	−0.0080 (15)	0.0094 (15)	−0.0048 (14)
O4	0.010 (2)	0.036 (3)	0.015 (2)	0.007 (2)	0.000	0.000
O5	0.0110 (15)	0.036 (2)	0.0117 (16)	−0.0044 (15)	−0.0006 (13)	−0.0003 (14)
O6	0.013 (2)	0.052 (4)	0.016 (2)	−0.004 (2)	0.000	0.000
O7	0.0223 (19)	0.0154 (17)	0.029 (2)	0.0031 (14)	0.0108 (16)	0.0055 (15)
O8	0.017 (2)	0.012 (2)	0.013 (2)	0.0099 (18)	0.000	0.000
B1	0.025 (3)	0.018 (3)	0.014 (2)	−0.007 (2)	0.004 (2)	−0.007 (2)
B2	0.016 (3)	0.015 (3)	0.006 (3)	0.002 (3)	0.000	0.000
B3	0.008 (2)	0.014 (2)	0.012 (2)	−0.0031 (18)	−0.0010 (18)	−0.0018 (19)
B4	0.013 (4)	0.024 (4)	0.016 (4)	−0.004 (3)	0.000	0.000

### Geometric parameters (Å, º)

**Table:**

Rb1—O3	2.980 (4)	Rb3—O5	3.105 (4)
Rb1—O3^i^	2.980 (4)	Rb3—O3^ii^	3.114 (4)
Rb1—O3^ii^	2.980 (4)	Rb3—O1^xi^	3.359 (5)
Rb1—O3^iii^	2.980 (4)	Rb3—B3	3.491 (5)
Rb1—O6^iv^	3.166 (6)	Rb3—B2	3.5061 (19)
Rb1—O6^v^	3.166 (6)	Rb3—B3^v^	3.603 (5)
Rb1—B2^iii^	3.610 (7)	Rb3—B4^v^	3.729 (5)
Rb1—B2	3.610 (7)	O1—B1	1.374 (7)
Rb1—Rb3	4.4556 (12)	O1—H1	0.9433
Rb1—Rb3^i^	4.4556 (12)	O2—B1	1.357 (6)
Rb1—Rb3^ii^	4.4556 (12)	O2—B3	1.495 (6)
Rb1—Rb3^iii^	4.4556 (12)	O3—B1	1.350 (7)
Rb2—O4^vi^	2.793 (5)	O3—B2	1.496 (5)
Rb2—O4	2.793 (5)	O4—B3^vii^	1.453 (5)
Rb2—O2^vii^	3.058 (4)	O4—B3	1.453 (5)
Rb2—O2^viii^	3.058 (4)	O5—B4	1.354 (5)
Rb2—O2^vi^	3.058 (4)	O5—B3	1.490 (6)
Rb2—O2	3.058 (4)	O6—B4	1.381 (9)
Rb2—B3	3.488 (5)	O6—H6	0.8200
Rb2—B3^vii^	3.488 (5)	O6—H6^vii^	0.8200
Rb2—B3^viii^	3.488 (5)	O7—B3	1.441 (6)
Rb2—B3^vi^	3.488 (5)	O7—B2	1.452 (5)
Rb2—Rb3^ix^	4.3609 (11)	O8—H8A	0.8500
Rb2—Rb3^x^	4.3609 (11)	O8—H8B	0.8500
Rb3—O7	2.816 (4)	O8—H8A^i^	0.8500
Rb3—O2^v^	2.963 (4)	O8—H8B^i^	0.8500
Rb3—O5^v^	2.974 (4)		
			
O3—Rb1—O3^i^	132.24 (13)	O3^ii^—Rb3—O1^xi^	158.35 (10)
O3—Rb1—O3^ii^	47.76 (13)	O7—Rb3—B3	23.42 (11)
O3^i^—Rb1—O3^ii^	180.00 (5)	O2^v^—Rb3—B3	154.19 (11)
O3—Rb1—O3^iii^	180.0	O5^v^—Rb3—B3	150.79 (11)
O3^i^—Rb1—O3^iii^	47.76 (13)	O5—Rb3—B3	25.25 (10)
O3^ii^—Rb1—O3^iii^	132.24 (13)	O3^ii^—Rb3—B3	67.90 (10)
O3—Rb1—O6^iv^	66.97 (7)	O1^xi^—Rb3—B3	91.11 (11)
O3^i^—Rb1—O6^iv^	66.97 (7)	O7—Rb3—B2	23.45 (12)
O3^ii^—Rb1—O6^iv^	113.03 (7)	O2^v^—Rb3—B2	151.80 (8)
O3^iii^—Rb1—O6^iv^	113.03 (7)	O5^v^—Rb3—B2	108.86 (9)
O3—Rb1—O6^v^	113.03 (7)	O5—Rb3—B2	67.94 (10)
O3^i^—Rb1—O6^v^	113.03 (7)	O3^ii^—Rb3—B2	25.24 (9)
O3^ii^—Rb1—O6^v^	66.97 (7)	O1^xi^—Rb3—B2	133.79 (11)
O3^iii^—Rb1—O6^v^	66.97 (7)	B3—Rb3—B2	42.74 (11)
O6^iv^—Rb1—O6^v^	180.0	O7—Rb3—B3^v^	146.09 (12)
O3—Rb1—B2^iii^	156.12 (7)	O2^v^—Rb3—B3^v^	23.88 (10)
O3^i^—Rb1—B2^iii^	23.88 (7)	O5^v^—Rb3—B3^v^	23.81 (10)
O3^ii^—Rb1—B2^iii^	156.12 (7)	O5—Rb3—B3^v^	155.00 (10)
O3^iii^—Rb1—B2^iii^	23.88 (7)	O3^ii^—Rb3—B3^v^	106.72 (10)
O6^iv^—Rb1—B2^iii^	90.0	O1^xi^—Rb3—B3^v^	92.62 (11)
O6^v^—Rb1—B2^iii^	90.0	B3—Rb3—B3^v^	166.87 (12)
O3—Rb1—B2	23.88 (7)	B2—Rb3—B3^v^	131.60 (10)
O3^i^—Rb1—B2	156.12 (7)	O7—Rb3—B4^v^	121.68 (14)
O3^ii^—Rb1—B2	23.88 (7)	O2^v^—Rb3—B4^v^	62.59 (13)
O3^iii^—Rb1—B2	156.12 (7)	O5^v^—Rb3—B4^v^	19.43 (12)
O6^iv^—Rb1—B2	90.0	O5—Rb3—B4^v^	165.50 (14)
O6^v^—Rb1—B2	90.0	O3^ii^—Rb3—B4^v^	74.88 (13)
B2^iii^—Rb1—B2	180.0	O1^xi^—Rb3—B4^v^	126.70 (14)
O3—Rb1—Rb3	63.01 (7)	B3—Rb3—B4^v^	141.27 (14)
O3^i^—Rb1—Rb3	135.79 (7)	B2—Rb3—B4^v^	99.31 (15)
O3^ii^—Rb1—Rb3	44.21 (7)	B3^v^—Rb3—B4^v^	39.47 (14)
O3^iii^—Rb1—Rb3	116.99 (7)	O7—Rb3—Rb2^v^	161.66 (8)
O6^iv^—Rb1—Rb3	119.50 (7)	O2^v^—Rb3—Rb2^v^	44.45 (7)
O6^v^—Rb1—Rb3	60.50 (7)	O5^v^—Rb3—Rb2^v^	65.47 (7)
B2^iii^—Rb1—Rb3	129.799 (15)	O5—Rb3—Rb2^v^	122.29 (7)
B2—Rb1—Rb3	50.201 (15)	O3^ii^—Rb3—Rb2^v^	135.72 (7)
O3—Rb1—Rb3^i^	135.79 (7)	O1^xi^—Rb3—Rb2^v^	64.64 (7)
O3^i^—Rb1—Rb3^i^	63.01 (7)	B3—Rb3—Rb2^v^	141.29 (9)
O3^ii^—Rb1—Rb3^i^	116.99 (7)	B2—Rb3—Rb2^v^	150.36 (11)
O3^iii^—Rb1—Rb3^i^	44.21 (7)	B3^v^—Rb3—Rb2^v^	50.87 (8)
O6^iv^—Rb1—Rb3^i^	119.50 (7)	B4^v^—Rb3—Rb2^v^	65.52 (11)
O6^v^—Rb1—Rb3^i^	60.50 (7)	O7—Rb3—Rb1	74.06 (8)
B2^iii^—Rb1—Rb3^i^	50.202 (15)	O2^v^—Rb3—Rb1	121.65 (7)
B2—Rb1—Rb3^i^	129.798 (15)	O5^v^—Rb3—Rb1	78.67 (7)
Rb3—Rb1—Rb3^i^	79.60 (3)	O5—Rb3—Rb1	105.16 (7)
O3—Rb1—Rb3^ii^	44.21 (7)	O3^ii^—Rb3—Rb1	41.86 (7)
O3^i^—Rb1—Rb3^ii^	116.99 (7)	O1^xi^—Rb3—Rb1	145.17 (7)
O3^ii^—Rb1—Rb3^ii^	63.01 (7)	B3—Rb3—Rb1	84.08 (9)
O3^iii^—Rb1—Rb3^ii^	135.79 (7)	B2—Rb3—Rb1	52.28 (12)
O6^iv^—Rb1—Rb3^ii^	60.50 (7)	B3^v^—Rb3—Rb1	99.81 (8)
O6^v^—Rb1—Rb3^ii^	119.50 (7)	B4^v^—Rb3—Rb1	60.50 (11)
B2^iii^—Rb1—Rb3^ii^	129.798 (14)	Rb2^v^—Rb3—Rb1	98.86 (3)
B2—Rb1—Rb3^ii^	50.202 (14)	B1—O1—Rb3^xii^	116.5 (4)
Rb3—Rb1—Rb3^ii^	100.40 (3)	B1—O1—H1	96.8
Rb3^i^—Rb1—Rb3^ii^	180.0	Rb3^xii^—O1—H1	78.5
O3—Rb1—Rb3^iii^	116.99 (7)	B1—O2—B3	120.9 (4)
O3^i^—Rb1—Rb3^iii^	44.21 (7)	B1—O2—Rb3^x^	120.5 (3)
O3^ii^—Rb1—Rb3^iii^	135.79 (7)	B3—O2—Rb3^x^	102.8 (2)
O3^iii^—Rb1—Rb3^iii^	63.01 (7)	B1—O2—Rb2	119.9 (3)
O6^iv^—Rb1—Rb3^iii^	60.50 (7)	B3—O2—Rb2	93.7 (3)
O6^v^—Rb1—Rb3^iii^	119.50 (7)	Rb3^x^—O2—Rb2	92.81 (9)
B2^iii^—Rb1—Rb3^iii^	50.202 (14)	B1—O3—B2	121.0 (4)
B2—Rb1—Rb3^iii^	129.798 (14)	B1—O3—Rb1	118.4 (3)
Rb3—Rb1—Rb3^iii^	180.0	B2—O3—Rb1	102.4 (3)
Rb3^i^—Rb1—Rb3^iii^	100.40 (3)	B1—O3—Rb3^ii^	122.9 (3)
Rb3^ii^—Rb1—Rb3^iii^	79.60 (3)	B2—O3—Rb3^ii^	92.20 (15)
O4^vi^—Rb2—O4	180.0	Rb1—O3—Rb3^ii^	93.93 (9)
O4^vi^—Rb2—O2^vii^	132.41 (6)	B3^vii^—O4—B3	124.6 (5)
O4—Rb2—O2^vii^	47.59 (6)	B3^vii^—O4—Rb2	106.2 (3)
O4^vi^—Rb2—O2^viii^	47.59 (6)	B3—O4—Rb2	106.2 (3)
O4—Rb2—O2^viii^	132.41 (6)	B4—O5—B3	121.2 (4)
O2^vii^—Rb2—O2^viii^	180.0	B4—O5—Rb3^x^	113.6 (4)
O4^vi^—Rb2—O2^vi^	47.59 (6)	B3—O5—Rb3^x^	102.5 (3)
O4—Rb2—O2^vi^	132.41 (6)	B4—O5—Rb3	123.3 (4)
O2^vii^—Rb2—O2^vi^	86.08 (13)	B3—O5—Rb3	92.0 (3)
O2^viii^—Rb2—O2^vi^	93.92 (13)	Rb3^x^—O5—Rb3	99.83 (10)
O4^vi^—Rb2—O2	132.41 (6)	B4—O6—Rb1^x^	128.7 (5)
O4—Rb2—O2	47.59 (6)	B4—O6—H6	125.4
O2^vii^—Rb2—O2	93.92 (13)	Rb1^x^—O6—H6	79.7
O2^viii^—Rb2—O2	86.08 (13)	B4—O6—H6^vii^	125.35 (13)
O2^vi^—Rb2—O2	180.0	Rb1^x^—O6—H6^vii^	79.74 (6)
O4^vi^—Rb2—B3	156.42 (9)	H6—O6—H6^vii^	102.7
O4—Rb2—B3	23.58 (9)	B3—O7—B2	123.7 (3)
O2^vii^—Rb2—B3	68.60 (11)	B3—O7—Rb3	105.6 (3)
O2^viii^—Rb2—B3	111.40 (11)	B2—O7—Rb3	106.0 (3)
O2^vi^—Rb2—B3	154.67 (10)	H8A—O8—H8B	107.7
O2—Rb2—B3	25.33 (10)	H8A—O8—H8A^i^	22.4
O4^vi^—Rb2—B3^vii^	156.42 (9)	H8B—O8—H8A^i^	93.7
O4—Rb2—B3^vii^	23.58 (9)	H8A—O8—H8B^i^	93.7
O2^vii^—Rb2—B3^vii^	25.33 (10)	H8B—O8—H8B^i^	76.3
O2^viii^—Rb2—B3^vii^	154.67 (10)	H8A^i^—O8—H8B^i^	107.7
O2^vi^—Rb2—B3^vii^	111.40 (11)	O3—B1—O2	124.1 (5)
O2—Rb2—B3^vii^	68.60 (11)	O3—B1—O1	117.8 (5)
B3—Rb2—B3^vii^	43.27 (17)	O2—B1—O1	118.1 (5)
O4^vi^—Rb2—B3^viii^	23.58 (9)	O7^ii^—B2—O7	113.4 (6)
O4—Rb2—B3^viii^	156.42 (9)	O7^ii^—B2—O3^ii^	110.79 (19)
O2^vii^—Rb2—B3^viii^	154.67 (10)	O7—B2—O3^ii^	107.1 (2)
O2^viii^—Rb2—B3^viii^	25.33 (10)	O7^ii^—B2—O3	107.1 (2)
O2^vi^—Rb2—B3^viii^	68.60 (11)	O7—B2—O3	110.79 (19)
O2—Rb2—B3^viii^	111.40 (11)	O3^ii^—B2—O3	107.5 (5)
B3—Rb2—B3^viii^	136.73 (17)	O7^ii^—B2—Rb3	150.8 (3)
B3^vii^—Rb2—B3^viii^	180.0	O7—B2—Rb3	50.53 (19)
O4^vi^—Rb2—B3^vi^	23.58 (9)	O3^ii^—B2—Rb3	62.57 (15)
O4—Rb2—B3^vi^	156.42 (9)	O3—B2—Rb3	101.8 (2)
O2^vii^—Rb2—B3^vi^	111.40 (11)	O7^ii^—B2—Rb3^ii^	50.52 (19)
O2^viii^—Rb2—B3^vi^	68.60 (11)	O7—B2—Rb3^ii^	150.8 (3)
O2^vi^—Rb2—B3^vi^	25.33 (10)	O3^ii^—B2—Rb3^ii^	101.8 (2)
O2—Rb2—B3^vi^	154.67 (10)	O3—B2—Rb3^ii^	62.57 (15)
B3—Rb2—B3^vi^	180.00 (9)	Rb3—B2—Rb3^ii^	155.0 (2)
B3^vii^—Rb2—B3^vi^	136.73 (17)	O7^ii^—B2—Rb1	123.3 (3)
B3^viii^—Rb2—B3^vi^	43.27 (17)	O7—B2—Rb1	123.3 (3)
O4^vi^—Rb2—Rb3^ix^	74.33 (8)	O3^ii^—B2—Rb1	53.8 (3)
O4—Rb2—Rb3^ix^	105.67 (8)	O3—B2—Rb1	53.8 (3)
O2^vii^—Rb2—Rb3^ix^	77.20 (7)	Rb3—B2—Rb1	77.52 (12)
O2^viii^—Rb2—Rb3^ix^	102.80 (7)	Rb3^ii^—B2—Rb1	77.52 (12)
O2^vi^—Rb2—Rb3^ix^	42.74 (7)	O7—B3—O4	112.5 (4)
O2—Rb2—Rb3^ix^	137.26 (7)	O7—B3—O5	108.2 (4)
B3—Rb2—Rb3^ix^	126.76 (8)	O4—B3—O5	110.8 (4)
B3^vii^—Rb2—Rb3^ix^	96.67 (8)	O7—B3—O2	111.3 (4)
B3^viii^—Rb2—Rb3^ix^	83.33 (8)	O4—B3—O2	107.2 (4)
B3^vi^—Rb2—Rb3^ix^	53.24 (8)	O5—B3—O2	106.9 (4)
O4^vi^—Rb2—Rb3^x^	105.67 (8)	O7—B3—Rb2	146.6 (3)
O4—Rb2—Rb3^x^	74.33 (8)	O4—B3—Rb2	50.3 (3)
O2^vii^—Rb2—Rb3^x^	102.80 (7)	O5—B3—Rb2	105.1 (3)
O2^viii^—Rb2—Rb3^x^	77.20 (7)	O2—B3—Rb2	61.0 (2)
O2^vi^—Rb2—Rb3^x^	137.26 (7)	O7—B3—Rb3	51.0 (2)
O2—Rb2—Rb3^x^	42.74 (7)	O4—B3—Rb3	149.7 (3)
B3—Rb2—Rb3^x^	53.24 (8)	O5—B3—Rb3	62.7 (2)
B3^vii^—Rb2—Rb3^x^	83.33 (8)	O2—B3—Rb3	102.9 (3)
B3^viii^—Rb2—Rb3^x^	96.67 (8)	Rb2—B3—Rb3	157.61 (16)
B3^vi^—Rb2—Rb3^x^	126.76 (8)	O7—B3—Rb3^x^	128.1 (3)
Rb3^ix^—Rb2—Rb3^x^	180.0	O4—B3—Rb3^x^	119.4 (3)
O7—Rb3—O2^v^	153.22 (11)	O5—B3—Rb3^x^	53.7 (2)
O7—Rb3—O5^v^	127.55 (10)	O2—B3—Rb3^x^	53.3 (2)
O2^v^—Rb3—O5^v^	47.64 (10)	Rb2—B3—Rb3^x^	75.88 (10)
O7—Rb3—O5	46.94 (10)	Rb3—B3—Rb3^x^	81.95 (10)
O2^v^—Rb3—O5	131.85 (10)	O5—B4—O5^vii^	124.3 (6)
O5^v^—Rb3—O5	169.84 (10)	O5—B4—O6	117.8 (3)
O7—Rb3—O3^ii^	46.81 (9)	O5^vii^—B4—O6	117.8 (3)
O2^v^—Rb3—O3^ii^	128.83 (9)	O5—B4—Rb3^x^	47.0 (3)
O5^v^—Rb3—O3^ii^	83.65 (10)	O5^vii^—B4—Rb3^x^	132.1 (5)
O5—Rb3—O3^ii^	92.98 (9)	O6—B4—Rb3^x^	90.9 (3)
O7—Rb3—O1^xi^	111.56 (10)	O5—B4—Rb3^xiii^	132.1 (5)
O2^v^—Rb3—O1^xi^	69.14 (10)	O5^vii^—B4—Rb3^xiii^	47.0 (3)
O5^v^—Rb3—O1^xi^	116.34 (10)	O6—B4—Rb3^xiii^	90.9 (3)
O5—Rb3—O1^xi^	65.88 (10)	Rb3^x^—B4—Rb3^xiii^	99.8 (2)
			
B2—O3—B1—O2	4.2 (8)	Rb2—O4—B3—O5	92.7 (4)
Rb1—O3—B1—O2	−123.3 (5)	B3^vii^—O4—B3—O2	−147.0 (4)
Rb3^ii^—O3—B1—O2	120.5 (5)	Rb2—O4—B3—O2	−23.6 (4)
B2—O3—B1—O1	−174.3 (5)	B3^vii^—O4—B3—Rb2	−123.5 (7)
Rb1—O3—B1—O1	58.2 (6)	B3^vii^—O4—B3—Rb3	40.4 (11)
Rb3^ii^—O3—B1—O1	−58.1 (6)	Rb2—O4—B3—Rb3	163.9 (5)
B3—O2—B1—O3	−3.0 (8)	B3^vii^—O4—B3—Rb3^x^	−89.9 (6)
Rb3^x^—O2—B1—O3	127.7 (5)	Rb2—O4—B3—Rb3^x^	33.5 (3)
Rb2—O2—B1—O3	−118.4 (5)	B4—O5—B3—O7	−107.7 (6)
B3—O2—B1—O1	175.6 (5)	Rb3^x^—O5—B3—O7	124.4 (3)
Rb3^x^—O2—B1—O1	−53.8 (6)	Rb3—O5—B3—O7	23.9 (3)
Rb2—O2—B1—O1	60.1 (6)	B4—O5—B3—O4	15.9 (7)
Rb3^xii^—O1—B1—O3	91.4 (5)	Rb3^x^—O5—B3—O4	−111.9 (4)
Rb3^xii^—O1—B1—O2	−87.2 (5)	Rb3—O5—B3—O4	147.5 (4)
B3—O7—B2—O7^ii^	−87.0 (4)	B4—O5—B3—O2	132.3 (5)
Rb3—O7—B2—O7^ii^	151.1 (2)	Rb3^x^—O5—B3—O2	4.5 (4)
B3—O7—B2—O3^ii^	150.5 (4)	Rb3—O5—B3—O2	−96.0 (3)
Rb3—O7—B2—O3^ii^	28.6 (4)	B4—O5—B3—Rb2	68.6 (6)
B3—O7—B2—O3	33.5 (6)	Rb3^x^—O5—B3—Rb2	−59.2 (2)
Rb3—O7—B2—O3	−88.4 (3)	Rb3—O5—B3—Rb2	−159.77 (14)
B3—O7—B2—Rb3	121.9 (5)	B4—O5—B3—Rb3	−131.6 (6)
B3—O7—B2—Rb3^ii^	−37.2 (9)	Rb3^x^—O5—B3—Rb3	100.53 (14)
Rb3—O7—B2—Rb3^ii^	−159.0 (5)	B4—O5—B3—Rb3^x^	127.9 (6)
B3—O7—B2—Rb1	93.0 (4)	Rb3—O5—B3—Rb3^x^	−100.53 (14)
Rb3—O7—B2—Rb1	−28.9 (2)	B1—O2—B3—O7	15.5 (6)
B1—O3—B2—O7^ii^	106.5 (5)	Rb3^x^—O2—B3—O7	−122.4 (3)
Rb1—O3—B2—O7^ii^	−119.1 (3)	Rb2—O2—B3—O7	143.9 (3)
Rb3^ii^—O3—B2—O7^ii^	−24.6 (3)	B1—O2—B3—O4	−107.8 (5)
B1—O3—B2—O7	−17.7 (6)	Rb3^x^—O2—B3—O4	114.3 (3)
Rb1—O3—B2—O7	116.7 (4)	Rb2—O2—B3—O4	20.6 (4)
Rb3^ii^—O3—B2—O7	−148.8 (3)	B1—O2—B3—O5	133.4 (5)
B1—O3—B2—O3^ii^	−134.4 (5)	Rb3^x^—O2—B3—O5	−4.5 (4)
Rb1—O3—B2—O3^ii^	0.000 (2)	Rb2—O2—B3—O5	−98.2 (3)
Rb3^ii^—O3—B2—O3^ii^	94.51 (12)	B1—O2—B3—Rb2	−128.3 (5)
B1—O3—B2—Rb3	−69.7 (5)	Rb3^x^—O2—B3—Rb2	93.72 (13)
Rb1—O3—B2—Rb3	64.69 (18)	B1—O2—B3—Rb3	68.4 (5)
Rb3^ii^—O3—B2—Rb3	159.20 (13)	Rb3^x^—O2—B3—Rb3	−69.56 (19)
B1—O3—B2—Rb3^ii^	131.1 (5)	Rb2—O2—B3—Rb3	−163.28 (12)
Rb1—O3—B2—Rb3^ii^	−94.51 (12)	B1—O2—B3—Rb3^x^	137.9 (5)
B1—O3—B2—Rb1	−134.4 (5)	Rb2—O2—B3—Rb3^x^	−93.72 (13)
Rb3^ii^—O3—B2—Rb1	94.51 (12)	B3—O5—B4—O5^vii^	−4.5 (11)
B2—O7—B3—O4	87.7 (6)	Rb3^x^—O5—B4—O5^vii^	118.2 (7)
Rb3—O7—B3—O4	−150.2 (3)	Rb3—O5—B4—O5^vii^	−121.1 (6)
B2—O7—B3—O5	−149.6 (4)	B3—O5—B4—O6	175.2 (6)
Rb3—O7—B3—O5	−27.6 (4)	Rb3^x^—O5—B4—O6	−62.1 (8)
B2—O7—B3—O2	−32.5 (6)	Rb3—O5—B4—O6	58.6 (8)
Rb3—O7—B3—O2	89.5 (3)	B3—O5—B4—Rb3^x^	−122.7 (6)
B2—O7—B3—Rb2	36.8 (8)	Rb3—O5—B4—Rb3^x^	120.7 (4)
Rb3—O7—B3—Rb2	158.8 (4)	B3—O5—B4—Rb3^xiii^	−64.7 (7)
B2—O7—B3—Rb3	−122.0 (5)	Rb3^x^—O5—B4—Rb3^xiii^	58.0 (5)
B2—O7—B3—Rb3^x^	−91.9 (5)	Rb3—O5—B4—Rb3^xiii^	178.7 (2)
Rb3—O7—B3—Rb3^x^	30.1 (4)	Rb1^x^—O6—B4—O5	90.1 (6)
B3^vii^—O4—B3—O7	90.4 (7)	Rb1^x^—O6—B4—O5^vii^	−90.1 (6)
Rb2—O4—B3—O7	−146.2 (3)	Rb1^x^—O6—B4—Rb3^x^	49.90 (10)
B3^vii^—O4—B3—O5	−30.8 (8)	Rb1^x^—O6—B4—Rb3^xiii^	−49.90 (10)

Symmetry codes: (i) *x*, *y*, −*z*+1; (ii) −*x*+2, −*y*+2, *z*; (iii) −*x*+2, −*y*+2, −*z*+1; (iv) *x*+1/2, −*y*+3/2, *z*−1/2; (v) −*x*+3/2, *y*+1/2, −*z*+3/2; (vi) −*x*+2, −*y*+1, −*z*+2; (vii) *x*, *y*, −*z*+2; (viii) −*x*+2, −*y*+1, *z*; (ix) *x*+1/2, −*y*+3/2, *z*+1/2; (x) −*x*+3/2, *y*−1/2, −*z*+3/2; (xi) *x*−1/2, −*y*+3/2, −*z*+3/2; (xii) *x*+1/2, −*y*+3/2, −*z*+3/2; (xiii) −*x*+3/2, *y*−1/2, *z*+1/2.

### Hydrogen-bond geometry (Å, º)

**Table:**

*D*—H···*A*	*D*—H	H···*A*	*D*···*A*	*D*—H···*A*
O8—H8*B*···O7^x^	0.85	2.25	3.046 (6)	155
O8—H8*B*···O4^x^	0.85	1.68	2.224 (7)	119
O8—H8*A*···O7^xiv^	0.85	1.70	2.231 (5)	118
O8—H8*A*···O4^xiv^	0.85	2.17	2.958 (7)	155
O6—H6···O1^xi^	0.82	1.86	2.670 (5)	167
O1—H1···O6^iv^	0.94	1.91	2.670 (5)	136

Symmetry codes: (iv) *x*+1/2, −*y*+3/2, *z*−1/2; (x) −*x*+3/2, *y*−1/2, −*z*+3/2; (xi) *x*−1/2, −*y*+3/2, −*z*+3/2; (xiv) *x*−1/2, −*y*+3/2, *z*−1/2.
